# Optimizing Drug Positioning in IBD: Clinical Predictors, Biomarkers, and Practical Approaches to Personalized Therapy

**DOI:** 10.3390/biomedicines14010191

**Published:** 2026-01-15

**Authors:** Irene Marafini, Silvia Salvatori, Antonio Fonsi, Giovanni Monteleone

**Affiliations:** 1 Gastroenterology Unit, Fondazione Policlinico Tor Vergata, Viale Oxford 81, 00133 Rome, Italy; irene.marafini@gmail.com (I.M.); silviasalvatori23@gmail.com (S.S.); antonio.fonsi@ptvonline.it (A.F.); 2 Department of Systems Medicine, University of Rome “Tor Vergata”, Via Montpellier, 1, 00133 Rome, Italy

**Keywords:** ulcerative colitis, Crohn’s disease, precision medicine, biologics, small molecules

## Abstract

Inflammatory Bowel Diseases (IBD), which include Crohn’s disease (CD) and ulcerative colitis (UC), are chronic, immune-mediated disorders marked by persistent and recurrent inflammation of the gastrointestinal tract. Over the past two decades, major advances in understanding the immunologic and molecular pathways that drive intestinal injury have transformed the therapeutic landscape. This progress has enabled the development of novel biologics and small-molecule agents that more precisely target dysregulated immune responses, thereby improving clinical outcomes and quality of life for many patients. Despite these therapeutic advances, IBD remains a highly heterogeneous condition. Patients differ widely in disease phenotype, progression, and response to specific treatments. Consequently, selecting the most effective therapy for an individual patient requires careful consideration of clinical features, molecular markers, and prior treatment history. The shift toward personalized, prediction-based treatment strategies aims to optimize the timing and choice of therapy, minimize unnecessary exposure to ineffective drugs, and ultimately alter the natural course of disease. In this review, we provide a comprehensive overview of current evidence guiding drug positioning in IBD, with particular emphasis on biologic therapies and small-molecule inhibitors. We also examine emerging biomarkers, clinical predictors of response, and real-world factors that influence therapeutic decision-making. Finally, we discuss the challenges and limitations that continue to hinder widespread implementation of personalized strategies, underscoring the need for further research to integrate precision medicine into routine IBD care.

## 1. Introduction

Crohn’s disease (CD) and ulcerative colitis (UC) are the primary forms of inflammatory bowel disease (IBD), affecting millions of people worldwide and presenting a significant burden to healthcare systems [1,2]. Both conditions involve inflammatory processes that cause varying degrees of intestinal damage, potentially leading to local complications and extra-intestinal manifestations. While the exact causes of CD and UC remain unclear, it is generally accepted that in genetically predisposed individuals, the immune response in the gut is triggered by a combination of environmental and host factors [3]. This response is characterized by the excessive production of inflammatory cytokines, which perpetuate and amplify the chronic inflammation [4,5]. This understanding has paved the way for the development of several therapeutic agents that have improved patient outcomes. Monoclonal antibodies and small molecules targeting specific inflammatory mediators have expanded the therapeutic options available [6,7]. Traditionally, treatment strategies followed a “step-up” approach, beginning with conventional drugs (e.g., mesalamine, corticosteroids) and escalating to biologics if necessary. However, this approach, which could delay effective control of inflammation and potentially contribute to disease progression, has increasingly been replaced by a “top-down” strategy [8]. This newer approach involves the early use of biologics or small molecules, especially for more aggressive forms of IBD. Despite these advancements, not all patients respond to these treatments, and some who initially benefit may become intolerant or develop secondary non-response over time [9]. Furthermore, both CD and UC exhibit clinical and tissue heterogeneity, which is believed to influence individual responses to treatment [10,11]. This variability has spurred research into identifying optimal candidates for advanced therapies and refining drug positioning. This review discusses current evidence on personalized treatment strategies for IBD, with a focus on biologics and small molecules, and explores the factors that may limit their effectiveness.

## 2. Step-Up and Top-Down Approaches

UC patients with distal colitis and a mild-to-moderate disease course can often be effectively managed with oral sulfasalazine or oral and/or rectal mesalamine (5-aminosalicylic acid, 5-ASA) at doses of 2.4–4.8 g/day. 5-ASA is well tolerated, induces remission, and helps maintain long-term remission, thereby reducing the risk of complications. However, its effectiveness in CD is limited. Antibiotics such as metronidazole and ciprofloxacin are occasionally used in CD, not for initial inflammation control but for specific complications, including abscesses, fistulas, or small intestinal bacterial overgrowth-related symptoms [12,13,14].

In the step-up approach, UC patients unresponsive or intolerant to 5-ASA can be treated with rectal or gut-release steroids with minimal systemic activity. For those with moderate-to-severe flare-ups or systemic extra-intestinal manifestations, oral corticosteroids are used. Intravenous corticosteroids are reserved for hospitalized patients with severe relapses. Steroid-dependent UC patients with moderate-to-severe disease may benefit from thiopurines (e.g., azathioprine) to maintain remission, while steroid-resistant patients are candidates for advanced therapies [12,13].

Recent interventional data strongly support early targeted treatment. The PROFILE trial, a biomarker-stratified randomized controlled trial of newly diagnosed active CD, demonstrated that a “top-down” approach using infliximab combined with an immunomodulator resulted in 79% sustained steroid-free, surgery-free remission at 48 weeks, compared to just 15% with the accelerated step-up approach. This strategy was associated with fewer flare-ups, surgeries, and serious adverse events. Interestingly, a 17-gene blood biomarker did not influence treatment outcomes, suggesting broad benefits of early combined immunosuppression [15]. Young age at onset, extensive disease, penetrating or perianal CD, and severe endoscopic inflammation are well-established predictors of a more aggressive disease course. These factors are associated with a higher risk of early complications, hospitalizations, and the need for surgery [16,17,18]. Therefore, patients exhibiting these high-risk features are ideal candidates for early biologic therapy to alter the disease’s natural progression, prevent structural damage, and optimize long-term outcomes [19].

## 3. Determinants of Therapeutic Positioning in IBD

**Figure 1:**
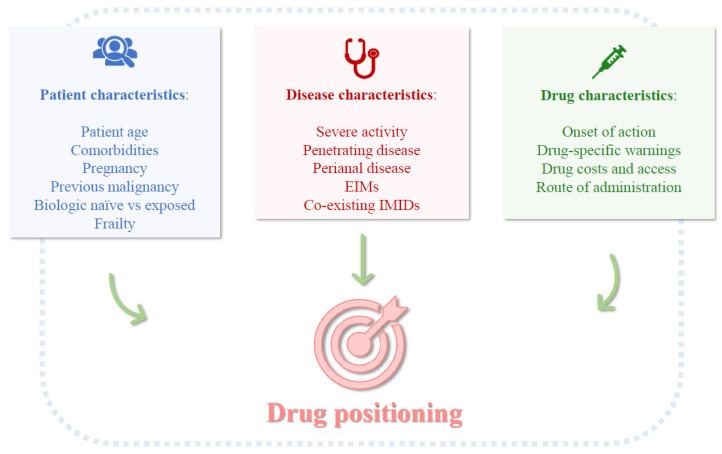
Schematic representation of the factors that may influence the positioning of a specific advanced therapy.

Drug positioning in IBD involves selecting the optimal advanced therapy for first-line treatment, tailored to individual patient profiles, disease activity, phenotype, and drug characteristics (Figure 1). Biologic-naïve patients consistently achieve higher remission rates across all drug classes, whereas those who have failed multiple biologics, particularly in CD, often show progressively lower response rates due to altered immune pathways or cumulative tissue damage [20,21]. This underscores the importance of therapeutic optimization or escalation, and in some cases, early surgical intervention.

Patient age plays a significant role in therapeutic choices. Many advanced therapies are not approved for pediatric patients, and Janus kinases (JAK) inhibitors are contraindicated in those over 65 years [6,7]. The presence of comorbidities, such as cardiovascular, ocular, or infectious diseases, can also limit the use of certain drugs. Additionally, extra-intestinal manifestations or co-existing immune-mediated conditions can influence drug selection. For example, anti-tumor necrosis factor (TNF) agents, JAK inhibitors, and anti-interleukin (IL) 23/p19 blockers are preferred in patients with significant joint or dermatologic involvement.

In addition to disease activity and previous treatment history, factors such as disease phenotype and reproductive considerations play a crucial role in guiding the selection of advanced therapies in IBD. These factors are not entirely addressed by algorithms based solely on efficacy. Disease phenotype is especially significant in CD. Patients presenting with penetrating or perianal manifestations tend to benefit most from early intervention with anti-TNF agents, which currently remain the only class of drugs supported by strong evidence for promoting fistula closure and preventing penetrating complications [22,23]. By contrast, patients with primarily inflammatory luminal disease, without evidence of strictures or fistulas, may experience similar clinical outcomes with alternative therapeutic classes. CD patients who have developed fibrostenotic complications exhibit limited response to medical therapies, highlighting the need for early, phenotype-guided treatment strategies aimed at preventing irreversible bowel damage rather than relying on late pharmacologic intervention [24]. Pregnancy and family planning represent additional, often underappreciated, factors influencing the choice and timing of therapy [25,26]. Anti-TNF agents have the most extensive safety data and are considered safe throughout gestation, with continuation into the third trimester advised for patients at high risk of disease relapse. Vedolizumab and ustekinumab have also shown reassuring real-world safety profiles, although long-term prospective data remain more limited. In contrast, small molecules, particularly JAK inhibitors and sphingosine-1-phosphate (S1P) modulators, are contraindicated during pregnancy because of teratogenic risk and should be avoided in women planning conception. Consequently, in patients of childbearing age, long-term treatment strategies should prioritize drugs with established reproductive safety, even when short-term efficacy appears similar across therapeutic classes.

Patients may also have preferences regarding the route of administration. Oral and subcutaneous medications are often preferred over intravenous therapies, which require clinic-based infusions. However, oral administration is associated with lower long-term adherence, particularly in chronic diseases requiring daily dosing, as patients may struggle to maintain compliance over time compared to infusional regimens administered under supervision. Therefore, treatment decisions should carefully balance efficacy, safety, and patient preferences, factoring in lifestyle and the capacity for adherence.

Recently, frailty status has emerged as an important consideration in IBD therapy, particularly in relation to the risk of anti-TNF-induced infections [27]. Frailty is defined as a multidimensional syndrome characterized by a decline in physical, cognitive, and functional domains, resulting in reduced physiological reserve and an impaired ability to cope with stressors [28]. The prevalence of frailty in IBD is notably high, even in younger patients, due to the chronic inflammatory burden, malnutrition, sarcopenia, and psychological distress commonly associated with the disease [27,29,30]. Although further prospective studies are needed to assess how frailty interacts with advanced therapies, its presence may influence treatment decisions and the overall management strategy.

From a health-economic perspective, drug costs and access are key factors in therapeutic positioning. Biosimilars have significantly lowered the cost of anti-TNF and anti-IL12/IL-23p40 inhibitors, making them the first-line biologic option in many public healthcare systems [31]. Meanwhile, newer agents such as IL-23 inhibitors, JAK inhibitors, and sphingosine-1-phosphate (S1P) modulators are often reserved for patients who have failed anti-TNFs. However, real-world evidence suggests that early use of high-efficacy therapies may be more cost-effective in the long run by reducing hospitalization, corticosteroid use, and surgery rates. Thus, the economic assessment should consider not just acquisition costs but the broader healthcare burden.

## 4. Positioning Advanced Therapies in IBD

### 4.1. Overview of the Treatment Selection

**Figure 2:**
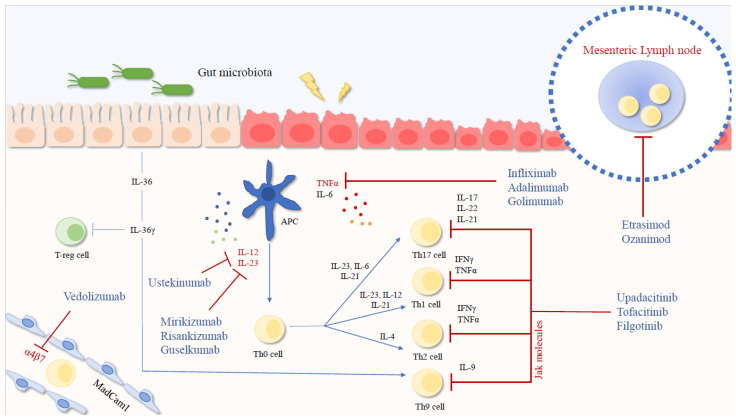
Overview of currently available advanced therapies for IBD and their molecular targets. Dysbiosis and multiple environmental triggers, together with genetic susceptibility, drive aberrant activation of both the innate and adaptive immune responses. Impaired APC activation contributes to intestinal epithelial injury through cytokine release, promoting the polarization of naïve Th0 cells into Th17, Th1, and Th2 subsets, while Th9 differentiation is primarily induced by IL-36γ produced by epithelial cells. The main molecular targets of advanced therapies include key pro-inflammatory cytokines, integrins, JAK molecules, and the S1P receptor, whose modulation leads to lymphocyte sequestration within peripheral lymphoid organs. IFNγ: Interferon-γ; TNFα: Tumor Necrosis Factor-α; IL: Interleukin; Jak Janus kinases.

Regardless of the first-line drug selected, the primary therapeutic goals in IBD are induction and maintenance of steroid-free remission, normalization of inflammatory markers (e.g., C-reactive protein, fecal calprotectin), and achieving mucosal and histological healing. Monoclonal antibodies used in IBD include anti-TNF agents, infliximab and adalimumab (for both CD and UC), and golimumab (for UC); the anti-α4β7 integrin antibody, vedolizumab (for both CD and UC); the anti-IL-12/23 p40 inhibitor, ustekinumab (for both CD and UC); and the IL-23 p19 inhibitors, guselkumab (for CD), mirikizumab (for UC), and risankizumab (for both CD and UC). Approved small molecules include JAK1 inhibitors like filgotinib (for UC) and upadacitinib (for CD and UC), the pan-JAK inhibitor tofacitinib (for UC), and S1P receptor modulators, such as etrasimod and ozanimod (for UC) (Figure 2).

Given the wide array of treatment options, selecting the most appropriate first-line therapy and determining subsequent therapeutic strategies can be complex. This complexity is further compounded by the considerable heterogeneity of IBD, which varies not only in clinical presentation and phenotype but also at the molecular level, influencing the underlying mechanisms of tissue damage and potentially impacting drug response. For instance, transcriptome analysis of biopsy samples from the inflamed colon of UC patients with similar clinical and endoscopic features has revealed two distinct molecular profiles, one of which is unresponsive to treatments with infliximab or vedolizumab [10]. Even within the same patient, the profile of effector cytokines targeted by advanced therapies can change over time, potentially explaining the varying patterns of treatment response loss, including primary versus secondary loss of response and immune versus non-immune mediated mechanisms [11]. Finally, it is important to consider that exposure to advanced therapies may significantly alter the profile of effector molecules in the gut. For example, in CD patients who are unresponsive to anti-TNF therapy, there is notable upregulation of IL-23/p19, IL-23 receptor (R), and IL-17A, along with an enhanced expansion of TNFR2^+^IL-23R^+^CD4^+^ T cells, which are resistant to anti-TNF-induced apoptosis [32]. This finding may help explain why certain drugs are more effective in specific patient subgroups. For example, ustekinumab, an inhibitor of the IL-12/23 p40 subunit, tends to be more effective than vedolizumab in patients previously exposed to anti-TNF therapy [33,34,35].

### 4.2. Evidence from Head-to-Head Clinical Trials

With the rapid expansion of available biologics and small molecules, direct comparative trials in IBD have become increasingly important. Head-to-head clinical trials remain the gold standard for determining the relative efficacy of different therapies and guiding therapeutic positioning [36]. So far, only a few head-to-head trials have compared advanced therapies for managing CD or UC. The first robust randomized trial to directly compare biologic agents with different mechanisms of action in moderate-to-severe IBD was the VARSITY trial, which evaluated vedolizumab versus adalimumab in UC [37]. At 52 weeks, a significantly higher percentage of patients in the vedolizumab group achieved clinical remission (31.3%) and endoscopic improvement (39.7%) compared to those in the adalimumab group (22.5% and 27.7%, respectively). By contrast, corticosteroid-free clinical remission was achieved in 12.6% of patients treated with vedolizumab versus 21.8% of those receiving adalimumab, though this difference did not reach statistical significance.

In the SEAVUE head-to-head trial, which compared ustekinumab and adalimumab in biologic-naïve patients with moderately-to-severely active CD, both ustekinumab and adalimumab monotherapies were highly effective, with no significant difference in the primary outcome, clinical remission at week 52, between the two drugs [38].

The SEQUENCE trial recently provided valuable insights into the comparative efficacy of IL-23 versus IL-12/23 inhibition in CD. This phase 3b, multicenter, randomized controlled trial compared risankizumab, a selective anti-IL-23 p19 monoclonal antibody, with ustekinumab in patients with moderate-to-severe CD who had failed or were intolerant to anti-TNF therapy. Risankizumab demonstrated noninferiority to ustekinumab in achieving clinical remission at week 24 (58.6% vs. 39.5%) and superiority in endoscopic remission at week 48 (31.8% vs. 16.2%). Additionally, all key secondary endpoints, including endoscopic response and steroid-free remission, consistently favored risankizumab, with a comparable safety profile. These findings suggest that selective IL-23 blockade may offer greater efficacy than IL-12/23 inhibition, particularly in the post-anti-TNF setting [39].

### 4.3. Insights from Network Meta-Analyses

Given the limited number of direct head-to-head clinical trials, researchers increasingly rely on network meta-analyses (NMAs) to make indirect comparisons among multiple therapeutic options. By integrating data from randomized controlled trials with a common comparator (usually placebo), NMAs estimate relative treatment efficacy and rank interventions through mixed treatment comparisons. While these analyses can support evidence-based treatment selection, they come with methodological challenges that require cautious interpretation. The reliability of NMAs depends on the assumptions of transitivity and consistency, which necessitate sufficient homogeneity across studies in terms of patient characteristics, disease severity, outcome definitions, and study design. In IBD, these assumptions are often only partially met, as clinical trials frequently differ in inclusion criteria (e.g., biologic-naïve vs. biologic-exposed patients), primary endpoints (e.g., clinical vs. endoscopic remission), follow-up duration, and concomitant medication use. Furthermore, variability in placebo response rates, geographic recruitment, and trial methodologies can introduce heterogeneity and bias, limiting the comparability and generalizability of NMA findings.

Barberio and colleagues conducted a network meta-analysis of 32 randomized controlled trials involving over 16,000 patients with UC. Their findings showed that upadacitinib (30 mg once daily) had the highest efficacy for both clinical remission and endoscopic improvement, followed by etrasimod and tofacitinib. Regarding endoscopic remission, vedolizumab (300 mg every 4 weeks) ranked highest across all patient groups, while for corticosteroid-free remission, guselkumab (200 mg every 4 weeks) achieved the top ranking [40].

A separate network meta-analysis assessed the comparative efficacy of biologic agents and small molecules for induction and maintenance of remission in luminal CD [41]. This analysis, encompassing 34 randomized controlled trials with over 14,000 patients, reported that infliximab (5 mg/kg) ranked highest for induction of clinical remission in patients with luminal CD. Among biologic-experienced patients, risankizumab (600 mg) showed the greatest efficacy for induction, while upadacitinib (30 mg daily) ranked highest for maintenance of remission. Safety outcomes were consistent with prior reports, with no significant differences in the incidence of serious adverse events across the therapies. In a separate analysis, Gorski et al. reported that, in addition to infliximab, guselkumab and mirikizumab were associated with higher probabilities of disease remission and improvements in quality of life, whereas small-molecule therapies demonstrated an intermediate efficacy profile [42].

The most recent AGA comparative analyses suggest that treatment selection should consider prior biologic exposure, which is the strongest predictor of therapeutic response. In biologic-naïve CD patients, most advanced therapies show similar efficacy. However, in biologic-experienced patients, significant differences emerge. IL-23 inhibitors, particularly risankizumab and guselkumab, demonstrate greater effectiveness than ustekinumab and vedolizumab for both induction and maintenance of remission [19,43]. These findings support an “early high-efficacy” strategy and a more personalized approach based on a patient’s therapeutic history.

From a clinical practice perspective, the lack of pragmatic sequencing trials means clinicians still rely on a combination of trial data, mechanistic rationale, patient-specific factors, and extraintestinal manifestations when positioning therapies [44]. Therefore, treatment decisions should not only account for efficacy and safety but also for convenience, comorbidities, risk of colectomy, and drug access, factors often not captured in clinical trials but crucial for real-world therapeutic positioning.

## 5. How Do We Measure Therapeutic Success?

The treat-to-target framework is now central to IBD management. The STRIDE-II consensus outlines clinical remission and endoscopic healing as primary long-term goals. Normalization of CRP and fecal calprotectin serve as intermediate targets, while histologic healing in UC and transmural healing in CD are considered adjunctive measures that reflect the depth of remission, though they are not formal targets [45]. This set of targets emphasizes the importance of tight monitoring and timely therapy optimization to prevent cumulative bowel damage.

Beyond endoscopy, intestinal ultrasound has become an increasingly valuable tool for real-time, radiation-free assessment of disease activity and early therapeutic response. It allows for the evaluation of bowel wall thickness, vascularity, and extramural complications, providing a more comprehensive view of inflammation compared to mucosal assessment alone and enabling earlier detection of treatment failure [46,47]. Additionally, the concept of disease clearance has emerged to describe a multidimensional state where patients achieve simultaneous symptomatic, biochemical, endoscopic, and, when applicable, transmural resolution of inflammation. In CD, transmural healing assessed by MRI or ultrasound is more strongly associated with reduced hospitalizations, fewer surgeries, and lower long-term disability than mucosal healing alone [48,49]. In UC, a combination of clinical, endoscopic, and histologic remission similarly reflects a deeper, more durable response [50,51]. Together, these evolving outcome measures underscore the shift toward composite targets that capture the full spectrum of inflammatory control, aiming not just for symptom relief, but for true modification of the natural history of IBD. Recent evidence suggests that this concept may need further refinement. Findings from the ERIca trial show that intestinal barrier healing, assessed through confocal endomicroscopy, outperforms both endoscopic and histologic remission in predicting long-term outcomes in UC and CD. It demonstrates significantly higher accuracy and predictive value for major adverse events [52]. These data suggest that achieving mucosal or even transmural healing may not fully capture the biological resolution of inflammation. Restoration of epithelial barrier integrity could represent a deeper level of therapeutic success. As monitoring tools and imaging technologies evolve, future treatment targets may extend beyond the current STRIDE definitions, incorporating functional healing domains that more accurately predict durable remission and true disease modification.

## 6. Therapeutic Drug Monitoring

Therapeutic drug monitoring (TDM) has become a cornerstone of treatment optimization in IBD, linking pharmacokinetics with therapeutic outcomes [53]. Drug concentrations vary based on disease phenotype, inflammatory burden, and therapeutic target, and maintaining adequate exposure within a defined therapeutic window correlates with superior endoscopic and histologic healing. While reactive TDM, performed after loss of response, is still the most common approach, multiple trials have shown that proactive TDM can prevent pharmacokinetic failure and improve long-term outcomes. The NOR-DRUM B and PAILOT trials demonstrated that scheduled monitoring during infliximab or adalimumab maintenance reduced antibody formation and secondary loss of response [54,55]. Proactive TDM also allows for early identification of underexposure during induction: infliximab levels > 20–25 µg/mL at week 2 and >15 µg/mL at week 6 are associated with higher mucosal healing rates in UC, while maintenance levels > 5 µg/mL predict long-term remission. Similar exposure–response relationships have been reported for vedolizumab (week 6 > 18 µg/mL), ustekinumab (week 8 > 3 µg/mL), and newer subcutaneous formulations.

The future of TDM lies in pharmacokinetic dashboards that integrate drug levels, antibodies, CRP, weight, and infusion timing to individualize dosing in real-time, as demonstrated in the PRECISION trial [56]. Point-of-care assays are also being developed to enable rapid therapeutic decisions and enhanced adherence monitoring. Thus, TDM has evolved from a reactive rescue tool to a proactive precision instrument bridging pharmacology with individualized care.

TDM is also essential for determining when therapeutic de-escalation, an integral part of precision-based care, can be considered [57]. De-escalation after anti-TNF intensification is feasible in about one-third of patients, with relapse rates of 30–38%. Best outcomes are seen in patients who achieve deep remission, defined by clinical, biochemical, and endoscopic healing [58]. Endoscopic and transmural remission correlate with the lowest relapse rates (10–25%), whereas elevated CRP or fecal calprotectin at the time of de-escalation predict failure. TDM helps identify candidates for de-escalation, as patients with supratherapeutic anti-TNF troughs can often reduce dosing without relapse risk. Conversely, those requiring prior dose escalation have a higher risk of relapse upon de-escalation and should be approached cautiously.

Real-world studies extend this evidence beyond anti-TNFs. In a cohort of 50 patients undergoing de-escalation of advanced combination therapy (biologic with or without JAK inhibitors), 76% maintained remission. Non-adherence and multiple prior biologic failures were the main risk factors for re-escalation [59], although these data should be taken with caution considering the retrospective nature of the study with a short median follow-up time. Gradual tapering was safer than abrupt withdrawal, and relapse was often responsive to re-treatment, with 80–100% of cases recapturing remission. The RAINBOWE study, the largest prospective cohort to date, followed over 700 IBD patients undergoing biologic or combination therapy de-escalation [60]. At one year, 72% maintained steroid-free remission, with most relapses occurring within six months. Predictors of sustained remission included deep mucosal healing, normal calprotectin levels, and therapeutic trough concentrations at the time of de-escalation. Importantly, 80–90% of relapsing patients recaptured remission after re-escalation with the same agent, confirming that loss of response is often reversible. The study also found no increase in adverse events, infections, or hospitalizations among those who successfully de-escalated, supporting the safety of gradual tapering in stable remission [60].

## 7. Molecular Profiling

Beyond pharmacokinetics, biomarker-based therapy is key to refining patient stratification in IBD. CRP and fecal calprotectin are validated, noninvasive tools for assessing inflammatory burden and guiding treatment escalation or de-escalation within the treat-to-target paradigm. However, no single biomarker has proven consistently useful due to variability across studies. Additionally, patients with similar clinical or endoscopic activity may display distinct molecular and immunologic profiles that influence therapeutic outcomes. For example, Czarnewski et al. demonstrated that UC patients with similar endoscopic severity could be segregated into two transcriptomic clusters (UC1 and UC2), each with markedly different responses to biologic therapy: 87% of UC1 patients failed infliximab or vedolizumab, while UC2 patients achieved mucosal healing [10], highlighting how molecular stratification can uncover subgroups undetectable by clinical assessment and form the basis for precision-guided therapies.

To further refine patient stratification, integrating multi-omic data, combining transcriptomic, proteomic, microbial, and metabolic information, is essential for defining biologically coherent disease subsets with predictable drug responsiveness. Pharmacogenetic testing already supports personalized treatment: TPMT and NUDT15 genotyping prevent thiopurine-induced myelotoxicity [61,62], and HLA-DQA1*05 variants are associated with heightened anti-TNF immunogenicity [63]. Genetic polymorphisms in IL23R, FasL, and caspase-9 have also shown moderate predictive value for treatment efficacy [64,65,66].

Transcriptomic profiling has identified reproducible molecular predictors of treatment response. Mucosal IL23A and IL22-related signatures correlate with responsiveness to IL-23 inhibitors [67], while high mucosal oncostatin M expression is linked to refractory phenotypes across biologic classes [68]. Th17/IL-23-driven transcriptional activity is associated with responsiveness to risankizumab or guselkumab [69]. Microbiome and metabolome studies also provide insights into patient stratification: Faecalibacterium prausnitzii and Roseburia inulinivorans (short-chain fatty acid producers) correlate with better outcomes on anti-TNF or vedolizumab therapy, while depletion of butyrate-producing taxa predicts resistance [70,71]. Metabolomic signatures enriched in butyrate and bile acid derivatives have similarly been linked to improved anti-TNF responsiveness.

A major advancement toward real-time personalization is molecular endoscopy, which combines fluorescently labeled antibodies with confocal endomicroscopy to visualize target engagement in vivo. Atreya et al. demonstrated that CD patients with high mucosal membrane-bound TNF expression had a 92% short-term response to infliximab, with significantly greater one-year mucosal healing [72]. Similarly, α4β7 integrin-positive mucosa predicted vedolizumab response, suggesting that imaging target availability can serve as a functional biomarker [73]. Advances in single-cell RNA sequencing, spatial transcriptomics, and AI-driven pattern recognition are complementing these techniques, enabling deep profiling of immune and stromal cell states that influence drug response or resistance.

Together, these multi-layered approaches, spanning pharmacokinetics, genetics, molecular profiling, microbiomics, and imaging, mark the shift from empiric therapy to precision-guided management in IBD. The integration of validated biomarkers with artificial intelligence (AI)-assisted clinical algorithms represents the next frontier, aiming to deliver “the right drug, at the right dose, to the right patient,” maximizing steroid-free remission while minimizing toxicity, cost, and therapeutic delay.

## 8. Precision Medicine Initiatives

Several large-scale precision medicine initiatives are driving the translation of molecular and pharmacologic discoveries into clinical practice. The PANTS study (Personalised Anti-TNF Therapy in Crohn’s Disease), the largest prospective anti-TNF cohort to date, followed over 1600 biologic-naïve patients for three years and provided important insights into long-term treatment dynamics [74]. Notably, only about one-third of patients maintained remission after three years, while two-thirds of initial responders eventually lost response. Multivariable analysis identified low week-14 drug concentrations as the strongest predictor of loss of response for both infliximab and adalimumab, with optimal thresholds of 6–10 mg/L for infliximab and 10–12 mg/L for adalimumab. Immunogenicity occurred in 44% of infliximab-treated and 20% of adalimumab-treated patients, particularly in HLA-DQA1*05 carriers and those not receiving concomitant immunomodulators. Early combination therapy or proactive dose optimization significantly prolonged drug persistence.

The IBD-Character consortium extends this approach through a multi-omics platform, integrating genomic, transcriptomic, proteomic, and microbiome data to define molecular endotypes linked to treatment response and disease course [75]. Similarly, the RISK and PREDICTS cohorts have identified early immune and microbial signatures that predict relapse or treatment resistance [76]. IBD Plexus, a large North American data ecosystem, integrates multi-omic profiles with real-world longitudinal data to validate predictive biomarkers across populations. The integration of AI and machine learning into these datasets is accelerating biomarker discovery and enabling real-time therapeutic predictions. AI-driven models are now being developed to anticipate response trajectories, optimize biologic sequencing, and predict adverse events, laying the foundation for clinical decision-support tools that can deliver truly personalized, bedside therapy. These efforts signal a paradigm shift from empirical drug selection to data-driven, individualized IBD care, where molecular profiling, pharmacogenetics, and digital prediction models converge to guide the right therapy at the right time for each patient.

## 9. Future Directions

The next frontier in IBD therapy involves the integration of multi-omics discovery, artificial intelligence, and mechanistically targeted interventions to provide truly personalized and potentially curative care. The integration of genomic, transcriptomic, proteomic, metabolomic, and microbiome data from large international consortia, such as IBD-Character, IBD Plexus, and PANTS, is refining our understanding of disease heterogeneity and enabling the identification of molecular types predictive of therapeutic response or resistance. These data-driven insights will allow clinicians to transition from empirical drug sequencing to biologically informed treatment selection and optimization.

Emerging biomarkers like oncostatin M, α4β7 integrin expression, IL-23-related gene signatures, and pharmacogenetic markers (e.g., HLA-DQA1*05) are showing promise in guiding therapeutic decisions. When combined with real-time therapeutic drug monitoring (TDM), these tools could enable more precise drug exposure management, minimize loss of response, and improve long-term remission durability.

Novel immune-cell-based therapies are also emerging for patients with multidrug-refractory disease. For example, a recent case reported by Müller et al. demonstrated that a single infusion of autologous CD19-directed CAR T cells led to complete clinical, endoscopic, and histologic remission of severe UC resistant to multiple biologics and small molecules [77]. This approach achieved profound and durable B-cell depletion in both blood and colonic tissue, highlighting the potential role of pathogenic B-cell subsets in otherwise treatment-resistant UC. While preliminary, these results suggest that cellular immunotherapy could represent a promising therapeutic avenue for selected patients, complementing conventional anti-cytokine or small-molecule therapies. However, further studies are needed to define optimal patient selection, mechanistic biomarkers, and long-term safety, particularly regarding infection and oncologic risks, before these therapies can be incorporated into mainstream practice [78].

## 10. Conclusions

While the promise of tailored medicine in IBD is compelling, several factors still limit its broad clinical application. Many biomarkers and multi-omics signatures under investigation have yet to be validated in large, independent, multicenter cohorts, and their effects are often modest, limiting their immediate utility in routine practice. Moreover, the technologies required are often slow, expensive, and not yet integrated into everyday clinical workflows. TDM and biomarker-based strategies hold potential but may vary across different drugs, disease phenotypes, and healthcare settings.

Access and equity remain significant challenges. The infrastructure, costs, and expertise required for precision medicine may widen the gap between specialized centers and community practices. Furthermore, the limitations of current drug positioning strategies are evident. Despite the availability of multiple mechanisms of action, comparative effectiveness data remain sparse, with only a few head-to-head trials offering direct guidance on sequencing therapies. Most recommendations still rely on indirect evidence or expert opinion. In real-world practice, treatment decisions are often influenced by cost considerations, which may force clinicians into suboptimal sequencing despite the biological rationale. Additionally, the lack of validated predictors for treatment durability means that therapeutic positioning remains reactive rather than truly proactive.

These challenges underscore that while drug positioning has improved, it is still far from a standardized or fully evidence-based process.

## Data Availability

No new data were created or analyzed in this study.

## References

[1] Dolinger M., Torres J., Vermeire S. (2024). Crohn’s disease. Lancet.

[2] Ungaro R., Mehandru S., Allen P.B., Peyrin-Biroulet L., Colombel J.F. (2017). Ulcerative colitis. Lancet.

[3] Monteleone G., Moscardelli A., Colella A., Marafini I., Salvatori S. (2023). Immune-mediated inflammatory diseases: Common and different pathogenic and clinical features. Autoimmun. Rev..

[4] Pallone F., Monteleone G. (1998). Interleukin 12 and Th1 responses in inflammatory bowel disease. Gut.

[5] Monteleone I., Pallone F., Monteleone G. (2011). Th17-related cytokines: New players in the control of chronic intestinal inflammation. BMC Med..

[6] Salvatori S., Marafini I., Fonsi A., Monteleone G. (2025). Advanced therapies targeting IL-23: Clinical outcomes in ulcerative colitis. Expert Opin. Biol. Ther..

[7] Salvatori S., Neri B., Marafini I., Brigida M., Monteleone G. (2023). Emerging oral drug options for ulcerative colitis. Expert Opin. Emerg. Drugs.

[8] Devlin S.M., Panaccione R. (2010). Evolving inflammatory bowel disease treatment paradigms: Top-down versus step-up. Med. Clin. N. Am..

[9] Yanai H., Hanauer S.B. (2011). Assessing response and loss of response to biological therapies in IBD. Am. J. Gastroenterol..

[10] Czarnewski P., Parigi S.M., Sorini C., Diaz O.E., Das S., Gagliani N., Villablanca E.J. (2019). Conserved transcriptomic profile between mouse and human colitis allows unsupervised patient stratification. Nat. Commun..

[11] Zorzi F., Monteleone I., Sarra M., Calabrese E., Marafini I., Cretella M., Sedda S., Biancone L., Pallone F., Monteleone G. (2013). Distinct profiles of effector cytokines mark the different phases of Crohn’s disease. PLoS ONE.

[12] Torres J., Bonovas S., Doherty G., Kucharzik T., Gisbert J.P., Raine T., Adamina M., Armuzzi A., Bachmann O., Bager P. (2020). ECCO Guidelines on Therapeutics in Crohn’s Disease: Medical Treatment. J. Crohn’s Colitis.

[13] Raine T., Bonovas S., Burisch J., Kucharzik T., Adamina M., Annese V., Bachmann O., Bettenworth D., Chaparro M., Czuber-Dochan W. (2022). ECCO Guidelines on Therapeutics in Ulcerative Colitis: Medical Treatment. J. Crohn’s Colitis.

[14] Troncone E., Monteleone G. (2017). The safety of non-biological treatments in Ulcerative Colitis. Expert Opin. Drug Saf..

[15] Noor N.M., Lee J.C., Bond S., Dowling F., Brezina B., Patel K.V., Ahmad T., Banim P.J., Berrill J.W., Cooney R. (2024). A biomarker-stratified comparison of top-down versus accelerated step-up treatment strategies for patients with newly diagnosed Crohn’s disease (PROFILE): A multicentre, open-label randomised controlled trial. Lancet Gastroenterol. Hepatol..

[16] Beaugerie L., Seksik P., Nion-Larmurier I., Gendre J.P., Cosnes J. (2006). Predictors of Crohn’s disease. Gastroenterology.

[17] Schwartz D.A., Loftus E.V., Tremaine W.J., Panaccione R., Harmsen W.S., Zinsmeister A.R., Sandborn W.J. (2002). The natural history of fistulizing Crohn’s disease in Olmsted County, Minnesota. Gastroenterology.

[18] Torres J., Mehandru S., Colombel J.F., Peyrin-Biroulet L. (2017). Crohn’s disease. Lancet.

[19] Lichtenstein G.R., Loftus E.V., Afzali A., Long M.D., Barnes E.L., Isaacs K.L., Ha C.Y. (2025). ACG Clinical Guideline: Management of Crohn’s Disease in Adults. Am. J. Gastroenterol..

[20] Lu X., Jarrett J., Sadler S., Tan M., Dennis J., Jairath V. (2023). Comparative efficacy of advanced treatments in biologic-naive or biologic-experienced patients with ulcerative colitis: A systematic review and network meta-analysis. Int. J. Clin. Pharm..

[21] Shehab M., Alrashed F., Alrashidi A., Hassan A., Ma C., Narula N., Jairat V., Regueiro M., Bessissow T. (2025). Network Meta-Analysis: Comparative Efficacy of Biologics and Small Molecules in the Induction and Maintenance of Remission in Crohn’s Disease. Aliment. Pharmacol. Ther..

[22] Shehab M., Alrashed F., Heron V., Restellini S., Bessissow T. (2023). Comparative Efficacy of Biologic Therapies for Inducing Response and Remission in Fistulizing Crohn’s Disease: Systematic Review and Network Meta-Analysis of Randomized Controlled Trials. Inflamm. Bowel Dis..

[23] Aboursheid T., Beran A., Hijazi M., Albuni M.K., Sawaf B., Guardiola J.J., Abdeljawad K., McDonald B.D., Rubin D.T. (2025). Comparison Between Adalimumab and Infliximab in Perianal Crohn’s Disease: A Systematic Review and Meta-Analysis. Gastro Hep Adv..

[24] Solitano V., Dal Buono A., Gabbiadini R., Wozny M., Repici A., Spinelli A., Vetrano S., Armuzzi A. (2023). Fibro-Stenosing Crohn’s Disease: What Is New and What Is Next?. J. Clin. Med..

[25] Mahadevan U., Seow C.H., Barnes E.L., Chaparro M., Flanagan E., Friedman S., Julsgaard M., Kane S., Ng S., Torres J. (2025). Global Consensus Statement on the Management of Pregnancy in Inflammatory Bowel Disease. J. Crohn’s Colitis.

[26] Mahadevan U., Seow C.H., Barnes E.L., Chaparro M., Flanagan E., Friedman S., Julsgaard M., Kane S., Ng S., Torres J. (2025). Global consensus statement on the management of pregnancy in inflammatory bowel disease. Gut.

[27] Salvatori S., Marafini I., Venuto C., Laudisi F., Neri B., Lavigna D., Franchin M., De Cristofaro E., Biancone L., Calabrese E. (2023). Frail Phenotype in Patients With Inflammatory Bowel Disease. Inflamm. Bowel Dis..

[28] Ananthakrishnan A.N. (2021). Frailty in Patients With Inflammatory Bowel Disease. Gastroenterol. Hepatol..

[29] Salvatori S., Marafini I., Franchin M., Lavigna D., Brigida M., Venuto C., Biancone L., Calabrese E., Giannarelli D., Monteleone G. (2023). Reversibility of Frail Phenotype in Patients with Inflammatory Bowel Diseases. J. Clin. Med..

[30] Kochar B., Orkaby A.R., Ananthakrishnan A.N., Ritchie C.S. (2021). Frailty in inflammatory bowel diseases: An emerging concept. Ther. Adv. Gastroenterol..

[31] Rezk M.F., Pieper B. (2020). Unlocking the Value of Anti-TNF Biosimilars: Reducing Disease Burden and Improving Outcomes in Chronic Immune-Mediated Inflammatory Diseases: A Narrative Review. Adv. Ther..

[32] Schmitt H., Billmeier U., Dieterich W., Rath T., Sonnewald S., Reid S., Hirschmann S., Hildner K., Waldner M.J., Mudter J. (2019). Expansion of IL-23 receptor bearing TNFR2+ T cells is associated with molecular resistance to anti-TNF therapy in Crohn’s disease. Gut.

[33] Yang H., Huang Z., Li M., Zhang H., Fu L., Wang X., Yang Q., He Y., Wu W., Jiang T. (2023). Comparative effectiveness of ustekinumab vs. vedolizumab for anti-TNF-naive or anti-TNF-exposed Crohn’s disease: A multicenter cohort study. EClinicalMedicine.

[34] Garcia M.J., Rivero M., Fernandez-Clotet A., de Francisco R., Sicilia B., Mesonero F., de Castro M.L., Casanova M.J., Bertoletti F., Garcia-Alonso F.J. (2024). Comparative Study of the Effectiveness of Vedolizumab Versus Ustekinumab After Anti-TNF Failure in Crohn’s Disease (Versus-CD): Data from the ENEIDA Registry. J. Crohn’s Colitis.

[35] Sharip M.T., Nishad N., Pillay L., Goordoyel N., Goerge S., Subramanian S. (2024). Ustekinumab or Vedolizumab after Failure of Anti-TNF Agents in Crohn’s Disease: A Review of Comparative Effectiveness Studies. J. Clin. Med..

[36] Pouillon L., Travis S., Bossuyt P., Danese S., Peyrin-Biroulet L. (2020). Head-to-head trials in inflammatory bowel disease: Past, present and future. Nat. Rev. Gastroenterol. Hepatol..

[37] Sands B.E., Peyrin-Biroulet L., Loftus E.V., Danese S., Colombel J.F., Toruner M., Jonaitis L., Abhyankar B., Chen J., Rogers R. (2019). Vedolizumab versus Adalimumab for Moderate-to-Severe Ulcerative Colitis. N. Engl. J. Med..

[38] Sands B.E., Irving P.M., Hoops T., Izanec J.L., Gao L.L., Gasink C., Greenspan A., Allez M., Danese S., Hanauer S.B. (2022). Ustekinumab versus adalimumab for induction and maintenance therapy in biologic-naive patients with moderately to severely active Crohn’s disease: A multicentre, randomised, double-blind, parallel-group, phase 3b trial. Lancet.

[39] Peyrin-Biroulet L., Chapman J.C., Colombel J.F., Caprioli F., D’Haens G., Ferrante M., Schreiber S., Atreya R., Danese S., Lindsay J.O. (2024). Risankizumab versus Ustekinumab for Moderate-to-Severe Crohn’s Disease. N. Engl. J. Med..

[40] Barberio B., Gracie D.J., Black C.J., Ford A.C. (2025). Network Meta-Analysis: Efficacy of Biological Therapies and Small Molecules as Maintenance Therapy in Ulcerative Colitis. Aliment. Pharmacol. Ther..

[41] Barberio B., Gracie D.J., Black C.J., Ford A.C. (2023). Efficacy of biological therapies and small molecules in induction and maintenance of remission in luminal Crohn’s disease: Systematic review and network meta-analysis. Gut.

[42] Gorski D., Lazo R.E.L., de Souza D.A., Borba H.H.L., Pontarolo R., Tonin F.S. (2025). Biological Therapy and Small Molecules for Adults With Crohn’s Disease: Systematic Review and Network Meta-Analysis. Pharmacotherapy.

[43] Singh S., Murad M.H., Yuan Y., Ananthakrishnan A.N., Click B., Syal G., Haydek J.P., Agrawal M., Kappelman M.D., Lewis J.D. (2025). Comparative Efficacy of Advanced Therapies for Management of Moderate-to-Severe Crohn’s Disease: 2025 AGA Evidence Synthesis. Gastroenterology.

[44] Yanofsky R., Rubin D.T. (2025). A practical approach to positioning therapies in ulcerative colitis. J. Can. Assoc. Gastroenterol..

[45] Turner D., Ricciuto A., Lewis A., D’Amico F., Dhaliwal J., Griffiths A.M., Bettenworth D., Sandborn W.J., Sands B.E., Reinisch W. (2021). STRIDE-II: An Update on the Selecting Therapeutic Targets in Inflammatory Bowel Disease (STRIDE) Initiative of the International Organization for the Study of IBD (IOIBD): Determining Therapeutic Goals for Treat-to-Target strategies in IBD. Gastroenterology.

[46] Yanai H., Feakins R., Allocca M., Burisch J., Ellul P., Iacucci M., Maaser C., Zilli A., Zidar N., Wilkens R. (2025). ECCO-ESGAR-ESP-IBUS Guideline on Diagnostics and Monitoring of Patients with Inflammatory Bowel Disease: Part 2. J. Crohn’s Colitis.

[47] Kucharzik T., Taylor S., Allocca M., Burisch J., Ellul P., Iacucci M., Maaser C., Baldin P., Bhatnagar G., Ben-Horin S. (2025). ECCO-ESGAR-ESP-IBUS Guideline on Diagnostics and Monitoring of Patients with Inflammatory Bowel Disease: Part 1. J. Crohn’s Colitis.

[48] Zorzi F., Rubin D.T., Cleveland N.K., Monteleone G., Calabrese E. (2023). Ultrasonographic Transmural Healing in Crohn’s Disease. Am. J. Gastroenterol..

[49] Zorzi F., Ghosh S., Chiaramonte C., Lolli E., Ventura M., Onali S., De Cristofaro E., Fantini M.C., Biancone L., Monteleone G. (2020). Response Assessed by Ultrasonography as Target of Biological Treatment for Crohn’s Disease. Clin. Gastroenterol. Hepatol. Off. Clin. Pract. J. Am. Gastroenterol. Assoc..

[50] Allocca M., Calabrese E. (2025). Ultrasound-guided strategies in ulcerative colitis: Early prediction and targeting. J. Crohn’s Colitis.

[51] Centanni L., Cicerone C., Fanizzi F., D’Amico F., Furfaro F., Zilli A., Parigi T.L., Peyrin-Biroulet L., Danese S., Allocca M. (2025). Advancing Therapeutic Targets in IBD: Emerging Goals and Precision Medicine Approaches. Pharmaceuticals.

[52] Rath T., Atreya R., Bodenschatz J., Uter W., Geppert C.E., Vitali F., Fischer S., Waldner M.J., Colombel J.F., Hartmann A. (2023). Intestinal Barrier Healing Is Superior to Endoscopic and Histologic Remission for Predicting Major Adverse Outcomes in Inflammatory Bowel Disease: The Prospective ERIca Trial. Gastroenterology.

[53] Irving P.M., Gecse K.B. (2022). Optimizing Therapies Using Therapeutic Drug Monitoring: Current Strategies and Future Perspectives. Gastroenterology.

[54] Syversen S.W., Goll G.L., Jorgensen K.K., Olsen I.C., Sandanger O., Gehin J.E., Warren D.J., Sexton J., Mork C., Jahnsen J. (2020). Therapeutic drug monitoring of infliximab compared to standard clinical treatment with infliximab: Study protocol for a randomised, controlled, open, parallel-group, phase IV study (the NOR-DRUM study). Trials.

[55] Assa A., Matar M., Turner D., Broide E., Weiss B., Ledder O., Guz-Mark A., Rinawi F., Cohen S., Topf-Olivestone C. (2019). Proactive Monitoring of Adalimumab Trough Concentration Associated With Increased Clinical Remission in Children With Crohn’s Disease Compared With Reactive Monitoring. Gastroenterology.

[56] Strik A.S., Lowenberg M., Mould D.R., Berends S.E., Ponsioen C.I., van den Brande J.M.H., Jansen J.M., Hoekman D.R., Brandse J.F., Duijvestein M. (2021). Efficacy of dashboard driven dosing of infliximab in inflammatory bowel disease patients; a randomized controlled trial. Scand. J. Gastroenterol..

[57] Gisbert J.P., Chaparro M. (2024). De-escalation of Biologic Treatment in Inflammatory Bowel Disease: A Comprehensive Review. J. Crohn’s Colitis.

[58] Gisbert J.P., Donday M.G., Riestra S., Lucendo A.J., Benitez J.M., Navarro-Llavat M., Barrio J., Morales-Alvarado V.J., Rivero M., Busquets D. (2025). Withdrawal of antitumour necrosis factor in inflammatory bowel disease patients in remission: A randomised placebo-controlled clinical trial of GETECCU. Gut.

[59] Saleh A.A., Waghela R., Amini S., Moskow J., Irani M., Fan C., Glassner K., Abraham B.P. (2025). A Guide to De-escalation of Combination Therapy in Inflammatory Bowel Disease: A Retrospective Cohort Study. Crohn’s Colitis 360.

[60] Rubin de Celix C., Ricart E., Martin-Arranz M.D., de Francisco R., Garcia-Alonso F.J., Mesonero F., Gomollon F., de Castro L., Ramos L., Garcia-Lopez S. (2025). Frequency and Effectiveness of Dose Escalation and De-Escalation of Biologic Therapy in Inflammatory Bowel Disease: The RAINBOW-IBD Study of ENEIDA. Aliment. Pharmacol. Ther..

[61] Deenen M.J., van Noordenburg A.J., Bouwens-Bijsterveld J., van Dijk M.A., Stapelbroek J.M., Derijks L.J.J., Gilissen L.P.L., Deiman B. (2024). Genetic association analysis and frequency of NUDT15*3 with thiopurine-induced myelosuppression in patients with inflammatory bowel disease in a large Dutch cohort. Pharmacogenom. J..

[62] Ribeiro A.C., Gerheim P., Chebli J.M.F., Nascimento J.W.L., de Faria Pinto P. (2023). The Role of Pharmacogenetics in the Therapeutic Response to Thiopurines in the Treatment of Inflammatory Bowel Disease: A Systematic Review. J. Clin. Med..

[63] Sazonovs A., Kennedy N.A., Moutsianas L., Heap G.A., Rice D.L., Reppell M., Bewshea C.M., Chanchlani N., Walker G.J., Perry M.H. (2020). HLA-DQA1*05 Carriage Associated With Development of Anti-Drug Antibodies to Infliximab and Adalimumab in Patients With Crohn’s Disease. Gastroenterology.

[64] Hlavaty T., Ferrante M., Henckaerts L., Pierik M., Rutgeerts P., Vermeire S. (2007). Predictive model for the outcome of infliximab therapy in Crohn’s disease based on apoptotic pharmacogenetic index and clinical predictors. Inflamm. Bowel Dis..

[65] Hlavaty T., Pierik M., Henckaerts L., Ferrante M., Joossens S., van Schuerbeek N., Noman M., Rutgeerts P., Vermeire S. (2005). Polymorphisms in apoptosis genes predict response to infliximab therapy in luminal and fistulizing Crohn’s disease. Aliment. Pharmacol. Ther..

[66] Jurgens M., Laubender R.P., Hartl F., Weidinger M., Seiderer J., Wagner J., Wetzke M., Beigel F., Pfennig S., Stallhofer J. (2010). Disease activity, ANCA, and IL23R genotype status determine early response to infliximab in patients with ulcerative colitis. Am. J. Gastroenterol..

[67] Nishioka K., Ogino H., Chinen T., Ihara E., Tanaka Y., Nakamura K., Ogawa Y. (2021). Mucosal IL23A expression predicts the response to Ustekinumab in inflammatory bowel disease. J. Gastroenterol..

[68] West N.R., Hegazy A.N., Owens B.M.J., Bullers S.J., Linggi B., Buonocore S., Coccia M., Gortz D., This S., Stockenhuber K. (2017). Oncostatin M drives intestinal inflammation and predicts response to tumor necrosis factor-neutralizing therapy in patients with inflammatory bowel disease. Nat. Med..

[69] Bourgonje A.R., Ungaro R.C., Mehandru S., Colombel J.F. (2025). Targeting the Interleukin 23 Pathway in Inflammatory Bowel Disease. Gastroenterology.

[70] Ananthakrishnan A.N., Luo C., Yajnik V., Khalili H., Garber J.J., Stevens B.W., Cleland T., Xavier R.J. (2017). Gut Microbiome Function Predicts Response to Anti-integrin Biologic Therapy in Inflammatory Bowel Diseases. Cell Host Microbe.

[71] Wang C., Gu Y., Chu Q., Wang X., Ding Y., Qin X., Liu T., Wang S., Liu X., Wang B. (2024). Gut microbiota and metabolites as predictors of biologics response in inflammatory bowel disease: A comprehensive systematic review. Microbiol. Res..

[72] Atreya R., Neumann H., Neufert C., Waldner M.J., Billmeier U., Zopf Y., Willma M., App C., Munster T., Kessler H. (2014). In vivo imaging using fluorescent antibodies to tumor necrosis factor predicts therapeutic response in Crohn’s disease. Nat. Med..

[73] Rath T., Bojarski C., Neurath M.F., Atreya R. (2017). Molecular imaging of mucosal alpha4beta7 integrin expression with the fluorescent anti-adhesion antibody vedolizumab in Crohn’s disease. Gastrointest. Endosc..

[74] Chanchlani N., Lin S., Bewshea C., Hamilton B., Thomas A., Smith R., Roberts C., Bishara M., Nice R., Lees C.W. (2024). Mechanisms and management of loss of response to anti-TNF therapy for patients with Crohn’s disease: 3-year data from the prospective, multicentre PANTS cohort study. Lancet Gastroenterol. Hepatol..

[75] Verstockt S., Torres J., Verstockt B. (2022). The Road to Prognostication? A Five-Protein Panel Predicting Disease Course in Inflammatory Bowel Disease. Gastroenterology.

[76] Porter C.K., Riddle M.S., Gutierrez R.L., Princen F., Strauss R., Telesco S.E., Torres J., Choung R.S., Laird R.M., Leon F. (2019). Cohort profile of the PRoteomic Evaluation and Discovery in an IBD Cohort of Tri-service Subjects (PREDICTS) study: Rationale, organization, design, and baseline characteristics. Contemp. Clin. Trials Commun..

[77] Muller F., Atreya R., Volkl S., Aigner M., Kretschmann S., Kharboutli S., Leppkes M., Sitte S., Strobel D., Hartmann A. (2025). CD19 CAR T-Cell Therapy in Multidrug-Resistant Ulcerative Colitis. N. Engl. J. Med..

[78] Marafini I., Salvatori S., Troncone E., Monteleone G. (2025). CD19 CAR T-Cell therapy for refractory ulcerative colitis. Gastroenterology.

